# A Wearable Activity Tracker Intervention With and Without Weekly Behavioral Support Emails to Promote Physical Activity Among Women Who Are Overweight or Obese: Randomized Controlled Trial

**DOI:** 10.2196/28128

**Published:** 2021-12-16

**Authors:** Melissa Black, Jennifer Brunet

**Affiliations:** 1 School of Human Kinetics Faculty of Health Sciences University of Ottawa Ottawa, ON Canada; 2 Institut du savoir Monfort Hôpital Monfort Ottawa, ON Canada; 3 Cancer Therapeutic Program Ottawa Hospital Research Institute Ottawa, ON Canada

**Keywords:** behavior change, motivation, obesity, physical activity, women, mobile phone

## Abstract

**Background:**

Physical activity (PA) plays a fundamental role in combating the current obesity epidemic; however, most women who are overweight or obese are generally physically inactive. Wearable activity tracker interventions can help increase the PA levels in this population. Supplementing such interventions with behavioral support emails may further improve their effectiveness, but this remains to be confirmed.

**Objective:**

This study aims to determine if adding behavioral support emails to a wearable activity tracker intervention can further increase PA levels among women who are overweight or obese in comparison to a wearable activity tracker–only intervention and a control condition.

**Methods:**

Women with a BMI ≥25 kg/m^2^ who were not meeting the Canadian PA guidelines for aerobic and strength training were randomized into 1 of 3 groups. Group 1 received 6 weekly behavioral support emails, a wearable activity tracker, and a copy of the Canadian PA guidelines. Group 2 received a wearable activity tracker and a copy of the Canadian PA guidelines, and group 3 (control condition) received a copy of the Canadian PA guidelines. Self-reported data for walking and moderate to vigorous intensity PA were collected preintervention (week 0; prerandomization), postintervention (7 weeks postrandomization), and at follow-up (21 weeks postrandomization) and analyzed as metabolic equivalent of task minutes per week. In addition, potential mechanisms of behavior change (ie, basic psychological needs satisfaction and motivational regulations) were assessed for within- and between-group differences at all 3 time points. Data were analyzed using nonparametric statistical tests.

**Results:**

A total of 49 women were recruited; data from 47 women (mean age 37.57 years, SD 11.78 years; mean BMI 31.69 kg/m^2^, SD 5.97 kg/m^2^) were available for analysis. Group 1 reported a significant increase in walking from preintervention to postintervention (*χ*^2^_2_=7.5; *P*=.02) but not in moderate to vigorous intensity PA (*P*=.24). Group 1 also reported significant increases in perceptions of competence from preintervention to follow-up (*χ*^2^_2_=7.6; *P*=.02) and relatedness from preintervention to follow-up (*χ*^2^_2_=8.7; *P*=.005). Increases in perceived autonomy were observed for group 2 (*χ*^2^_2_=7.0) and group 3 (*χ*^2^_2_=10.6). There were no significant changes in the motivational regulations within the groups. The difference between the groups was not significant for any outcome variable.

**Conclusions:**

The results suggest that adding behavioral support emails to a wearable activity tracker intervention may help to increase time spent walking and perceptions of competence and relatedness for PA among women who are overweight or obese.

**Trial Registration:**

ClinicalTrials.gov NCT03601663; http://clinicaltrials.gov/ct2/show/NCT03601663

## Introduction

### Background

According to the World Health Organization, obesity is a major risk factor for serious conditions, such as diabetes, certain cancers, and heart diseases [1]. In North America, the prevalence of people who are overweight or obese is a public health concern reported to affect approximately 64% and 28% of the population, respectively [2]. Regular engagement in physical activity (PA) helps with weight management and can reduce the risk of developing certain health conditions associated with obesity [3]. However, large community-based surveys show that only 16% of adults living in Canada meet the current PA guidelines of 150 minutes of moderate to vigorous intensity aerobic PA per week [4], with lower rates being observed among women who are overweight or obese [5]. Therefore, there is a critical need to promote PA among women living in Canada, especially those who are overweight or obese, because it could help to reduce their risk of developing several health conditions, lower their risk of all-cause mortality, and offer them a better quality of life [6].

Previous interventions aimed at increasing PA levels among women who are overweight or obese have shown promising results [7-11], with many of these interventions being delivered face-to-face. Advancements in technology provide the opportunity to build on existing knowledge and develop PA behavior change interventions that are less time- and resource-intensive and more accessible for those who face barriers to attending face-to-face programming (eg, limited transportation options, rural communities, or anxiety about attending in person), which may allow more women to increase their PA levels. Technologies including email and messaging platforms allow specialists to deliver interventions and share well-established behavior change techniques with participants to promote PA, whereas activity tracking devices enable users to self-monitor their PA behavior and make changes accordingly. Several studies have shown that providing participants with a wearable activity tracker to self-monitor their PA behavior is associated with increases in PA [12,13]. Cadmus-Bertram et al [11] observed a 62-minute per week increase in moderate to vigorous intensity PA immediately following a 4-week intervention that provided women who were overweight or obese with a wearable activity tracker and an instructional session. Accordingly, several interventions for which efficacy has been demonstrated to increase PA levels, now provide participants with a wearable activity tracker [12,13].

Although wearable activity tracker interventions may help to increase PA levels initially, researchers have noted a decrease in PA levels following initial exposure to the device [14-16] and a lack of evidence regarding the effectiveness of wearable activity tracker use beyond the initial intervention phase [12]. Moreover, some studies have found that using a wearable activity tracker may undermine autonomous motivation for PA and associated processes [17-19]. Specifically, Kerner and Goodyear [18] found that providing participants with a wearable activity tracker can decrease basic psychological needs satisfaction and autonomous motivation for PA. Mendoza et al [19] found that providing participants with a wearable activity tracker can increase introjected motivation—a controlled form of motivation in which people behave to avoid feelings of guilt or enhance feelings of pride [20]. This is critically important because autonomous motivation is a significant and robust predictor of PA behavior and is associated with PA adherence, whereas controlled motivation is a neutral or negative predictor of long-term PA engagement [21]. Teaching other effective strategies that are based on relevant literature may help augment the short-term benefits of self-monitoring to increase PA by developing autonomous motivation to ensure that the changes are sustained over time [22,23]. Accordingly, it is possible that participants combining a wearable activity tracker with a theory-based behavioral intervention that targets core predictors of PA (eg, psychological needs satisfaction, motivation) may optimize increases in PA levels [24,25].

Harnessing technology to provide self-directed materials explaining effective behavior change techniques that align with contemporary theories of health behaviours could help to further increase and sustain PA levels. Email is a common tool for delivering self-directed materials within interventions. Not only is emailing free and familiar to most adult women, it also provides the opportunity to access the materials when it is suitable to them. Self-determination theory is a suitable theory to guide the development of such emails because it provides a powerful framework for explaining women’s PA behavior [10,26,27] and has previously been used to develop effective interventions among women who are overweight or obese [7-10]. Self-determination theory is a macrotheory of human motivation, in which motivation exists along a continuum from amotivation (ie, complete lack of motivation) through controlled motivation (ie, engagement in behavior for external reasons including rewards, pride, or guilt) to autonomous motivation (ie, engagement in behavior for its own sake) [20,27].More autonomous forms of motivation are more positively associated with PA behavior, with the most autonomous form (ie, intrinsic motivation) being the most predictive of long-term PA adherence [21,26]. The use of motivational and behavior change techniques within interventions can be used to enhance autonomous motivation [23] and elicit behavior changes [20] by fostering perceptions of autonomy, competence, and relatedness. Interventionists can deliver many of these techniques to participants over email; for example, they can provide participants with choices, encourage experimentation, and teach strategies for goal setting, self-monitoring, and addressing obstacles.

In addition, much of the literature examining interventions to promote PA has focused on increasing moderate to vigorous intensity PA. Few studies have focused on increasing low-intensity PA (eg, low-intensity walking) despite the growing evidence suggesting that it may have significant health benefits [28,29] and that it is often rated as more enjoyable and more pleasant in low-active women who are obese [30,31]. Walking, regardless of intensity, may also be easier to integrate into one’s everyday routines than other types of PA (eg, by replacing some driving with walking; going on social walks with a friend, spouse, or child; taking a lunchtime walk; parking further away from stores). Thus, interventions aimed at promoting walking may help increase PA levels in low-active women who are overweight or obese.

Drawing on self-determination theory [20,27] and previous research [18,19,32], the aim of this randomized controlled trial is to determine whether women who received a multicomponent intervention would increase their PA levels more than women who received fewer components. The multicomponent intervention consisted of 6 weekly autonomy-supportive emails designed to increase perceptions of competence, autonomy, and relatedness as well as autonomous motivation for walking and moderate to vigorous intensity PA; a wearable activity tracker to facilitate self-monitoring; and a paper copy and verbal explanation of the Canadian PA guidelines to establish a target for behavior change (group 1) as compared with receiving a wearable activity tracker to facilitate self-monitoring and a paper copy and verbal explanation of the Canadian PA guidelines (group 2) or a paper copy and verbal explanation of the Canadian PA guidelines only (group 3).

### Objective

The aim of this study is to assess changes in PA levels over time within each group and to determine if there were significant differences in changes in PA levels between the groups. A secondary objective is to explore changes in PA-related basic psychological needs satisfaction and motivational regulations within and between groups to gain more insight into any observed changes in PA.

## Methods

### Study Design

This study was a 3-arm parallel group randomized controlled trial featuring a 6-week intervention designed to increase PA levels among low-active women who were overweight or obese. The study was conducted in Ontario, Canada. The primary outcome of the trial was PA, and the secondary outcomes were PA-related basic psychological needs satisfaction and motivational regulations. Data were collected at preintervention (prerandomization; week 0), postintervention (week 7), and at follow-up (week 21) using a combination of self-report questionnaires and direct measurements. The reporting of this study is in accordance with the CONSORT 2010 statement [33] and the CONSORT guidelines for eHealth interventions [34]. This trial was registered at ClinicalTrials.gov (NCT03601663) on July 26, 2018, and was approved by the institutional review board at the University of Ottawa (H-06-18-437). All participants provided informed consent digitally through a web-based form.

### Recruitment and Study Sample

A convenience sample of women was recruited between September 2018 and March 2019 by advertising through social media (ie, Facebook), web-based boards (ie, Kijiji, Craigslist, or local classifieds), and posters in publicly accessible areas (ie, community centers or physician’s offices). Advertisements encouraged women to contact the research team for further information and eligibility screening.

Women were eligible if they met the following inclusion criteria: (1) identified as female; (2) aged 18-65 years; (3) BMI ≥25 kg/m^2^; (4) could read and write in English; (5) answered *no* to the question: *Do you have any health concerns that could prevent you from safely engaging in PA?*; (6) were not pregnant or lactating; (7) reported engaging in <150 minutes of moderate to vigorous intensity PA and strength or resistance training (eg, free weights, weight machines, resistance bands, and exercises using body weight) <2 times per week; (8) had access to internet and an active email account; (9) had not used a wearable activity tracking device in the past 12 months; and (10) lived <50 km from the University of Ottawa.

### Data Collection

Eligible participants were informed about all relevant aspects of the study before enrolling and then the digital consent was obtained. After providing consent, they were directed to a web-based platform (ie, SurveyMonkey) to complete the baseline questionnaires. The questionnaires were designed to collect sociodemographic information, health status, self-reported PA, basic psychological needs satisfaction for PA, and motivational regulations for PA. Once participants completed the questionnaires, they were invited to meet with the first author at the location of their preference, either their home or the University of Ottawa, to measure their height, weight, body mass, and body composition. After the measurements were taken and recorded, the first author opened an opaque envelope revealing the participants’ group allocation. Subsequent questionnaires were completed on the web by participants at postintervention (week 7) and at follow-up (week 21).

### Randomization

The randomization sequence was generated by an independent researcher using permuted blocks of 3 and 6 using a web-based randomization software program (Sealed Envelope Ltd, 2017). It was not possible to blind participants or the researchers because of the nature of the intervention and their role in delivering the intervention, respectively.

### Intervention Groups

#### Group 1

Participants randomized to group 1 received a paper copy and brief verbal explanation of the Canadian PA guidelines for adults aged 18-64 years. They also received a Polar A300 activity monitor with a charging cable and access to the Polar Flow web and smartphone apps for the duration of the 6-week intervention. All materials were provided when they met with the first author for the baseline assessment. They were instructed to wear the device on their wrist daily during waking hours for the 6-week intervention period, except when swimming or bathing, beginning the day following the baseline assessment. The first author provided instructions on how to navigate the device and assisted participants in syncing the device with their smartphone and/or computer so that they could review their PA data in greater detail.

In addition, during the 6-week intervention, participants received standardized emails from the first author on a weekly basis. The emails featured established motivational and behavior change techniques that align with self-determination theory and were written in a noncontrolling language [23,35] to enhance perceptions of autonomy (ie, perceived control over one’s actions), competence (ie, perceived mastery of tasks and skills), and relatedness (ie, perceived belonging and connection to others) and in turn enhance autonomous motivation for PA [36,37]. Key techniques included goal setting, action planning, contingency planning, and self-monitoring. Other recurring themes throughout the emails included learning from trial and error, focusing on making small changes, choosing enjoyable activities, and aligning plans with personal beliefs and values. A detailed overview of the contents and techniques included in the emails is provided in Multimedia Appendix 1.

#### Group 2

Participants randomized to group 2 received a paper copy and brief verbal explanation of the Canadian PA guidelines for adults aged 18-64 years and a Polar A300 activity monitor when they met with the first author at week 0. Group 2 was a comparison arm for testing if the combined intervention group 1 received was more effective than providing people with a wearable activity tracker alone, as few studies have isolated the effect of this component [38,39]. After completing the questionnaires at follow-up, group 2 participants were provided with a copy of the weekly emails to thank them for their participation in the study.

#### Group 3

Participants randomized to group 3 received a paper copy and brief verbal explanation of the Canadian PA guidelines for adults aged 18-64 years when they met with the first author at week 0 but no further treatment as group 3 represented the control condition. After completing the questionnaires at follow-up, group 3 participants were provided with a copy of the weekly emails to thank them for their participation in the study.

### Sample Size Determination

A priori, the target sample size was estimated using G*Power [40] to ensure sufficient power for the primary outcome of the total metabolic equivalent of task (MET)-minutes per week of PA. Using a mixed repeated-measures analysis of variance with 3 groups and 3 repeated measures, the target sample size was 36 participants, assuming an effect size of 0.25, α of .05, power of 0.80, and a correlation coefficient of among repeated measures of 0.50. These assumptions were made based on findings from a meta-analysis of pedometer-based PA interventions [41] and other interventions with overlapping features developed to promote PA in similar populations [11,42].

### Measures

#### Sociodemographic and Health Characteristics

Sociodemographic and health information were collected from participants before the intervention. Sociodemographic measures included age, marital status, race, highest level of education attained, number of children and their age, annual household income, and employment status. Health measures included self-reported history of chronic diseases, smoking history, and self-rated health. Self-rated health was measured using the first question of the 36-item Short Form Health Survey [43], which asks, “In general, how would you say your health is*?*” and provides 5 response categories: (1) *excellent*, (2) *very good*, (3) *good*, (4) *fair*, and (5) *poor*. Self-reported health was reassessed postintervention.

#### Anthropometrics

The height (m), body mass (kg), body composition, and waist circumference (cm) of the participants were measured preintervention and postintervention without shoes and with light clothes. Body mass and composition were measured using a hospital-grade body weight scale (TBF 300A, Tanita Corporation of America Inc). Height was measured using a portable wall-mounted height rod (HR-200, Tanita Corporation of America Inc). Waist circumference was measured over participants’ clothing with a measuring tape midway between the 10th rib and the top of the iliac crest. Before the measurements, participants were asked to refrain from the following: (1) drinking alcohol or engaging in moderate to vigorous intensity PA for 12 hours before meeting with the first author, (2) eating or drinking for 3 hours before the meeting, and (3) eating *excessively* or *restrictively* within 24 hours of the meeting [44].

#### PA Behavior

PA behavior (primary outcome) was assessed at all 3 time points using the International Physical Activity Questionnaire (IPAQ) Short Form. Participants were asked to report the number of days and average duration over the past week that they engaged in sedentary behaviors, walking, and moderate to vigorous intensity PA. The number of days was multiplied by the average duration to estimate the number of minutes per week for each category. Scores for vigorous, moderate, and walking activities were multiplied by 8.0, 4.0, and 3.3, respectively, to calculate the total number of MET minutes per week, which reflects the amount of energy expended in each category throughout the week. The scores for moderate and vigorous intensity PA were then summed to calculate moderate to vigorous intensity PA. Both moderate to vigorous intensity PA and walking MET minutes per week were analyzed as outcome variables. Scores on the IPAQ Short Form have demonstrated good reliability and validity for use in adult populations [45].

#### PA-Related Basic Psychological Needs Satisfaction

Basic psychological needs satisfaction in relation to PA (secondary outcome) was measured at all 3 time points using the Psychological Need Satisfaction in Exercise (PNSE) scale [46]. The PNSE scale consists of 18 statements that were used to calculate 3 subscale scores, which measure perceived autonomy, competence, and relatedness for exercise. All items were rated using a 6-point Likert scale ranging from (1) *false* to (6) *true*, wherein lower scores represent less needs satisfaction. For this study, the scale was modified by replacing the word *exercise* with *physical activity*. Internal reliability coefficients for each subscale of the PNSE in this study are presented in Multimedia Appendix 2.

#### Motivational Regulations for PA

Motivational regulations for PA (secondary outcome) were assessed at all 3 time points using the Behavioral Regulation in Exercise Questionnaire, version 3 (BREQ-3) [47,48]. The BREQ-3 includes 24 items divided into 6 subscales assessing all 6 motivational regulations; each motivational regulation was assessed as a separate outcome in this study given the limitations associated with using a combined score [49]. Participants were asked to respond to each item using a 5-point Likert scale ranging from (0) *not true for me* to (4) *very true for me*, wherein lower scores represent less of that motivational regulation. For this study, the scale was modified by replacing the word *exercise* with *physical activity*. The internal reliability coefficients of the BREQ-3 in this study are presented in Multimedia Appendix 2.

### Statistical Analysis

Data were analyzed using SPSS Statistics (version 26; IBM Corporation) following intent-to-treat principles in which data from all participants who were randomized were analyzed. Initially, all data were screened for missingness, outliers, and normality. Item-level missing data were imputed by calculating the mean score of the subscale to which the missing item belonged; person-level missing data (ie, data missing because of participant attrition) was imputed by replacing the missing subscale scores with the last observation. Descriptive statistics were calculated for all variables. Pairwise correlations were estimated between moderate to vigorous intensity PA, walking, BMI, age, education, and depressive symptoms at baseline to identify any potential covariates. No correlations were statistically significant, and thus were not included in the analyses.

The Shapiro-Wilk test was used to check for normality, as recommended for sample sizes <50. As the data were found to be nonnormally distributed, the Friedman test (ie, the nonparametric equivalent to the 2-way analysis of variance) was used to test for significant differences in median values between time points within groups. Pairwise comparisons were performed using the Wilcoxon signed-rank test with a Bonferroni correction for multiple comparisons to locate differences. The Kruskal-Wallis H test was used to assess for differences in change scores between groups. For these tests, change scores were calculated between the time points by subtracting the later values (eg, postintervention) from the former values (eg, preintervention). Statistically significant differences between the groups were further analyzed with post hoc analyses, namely pairwise comparisons using the Dunn procedure with a Bonferroni correction for multiple comparisons. Bonferroni adjusted *P* values <.05 were considered statistically significant.

## Results

### Participants

**Figure 1:**
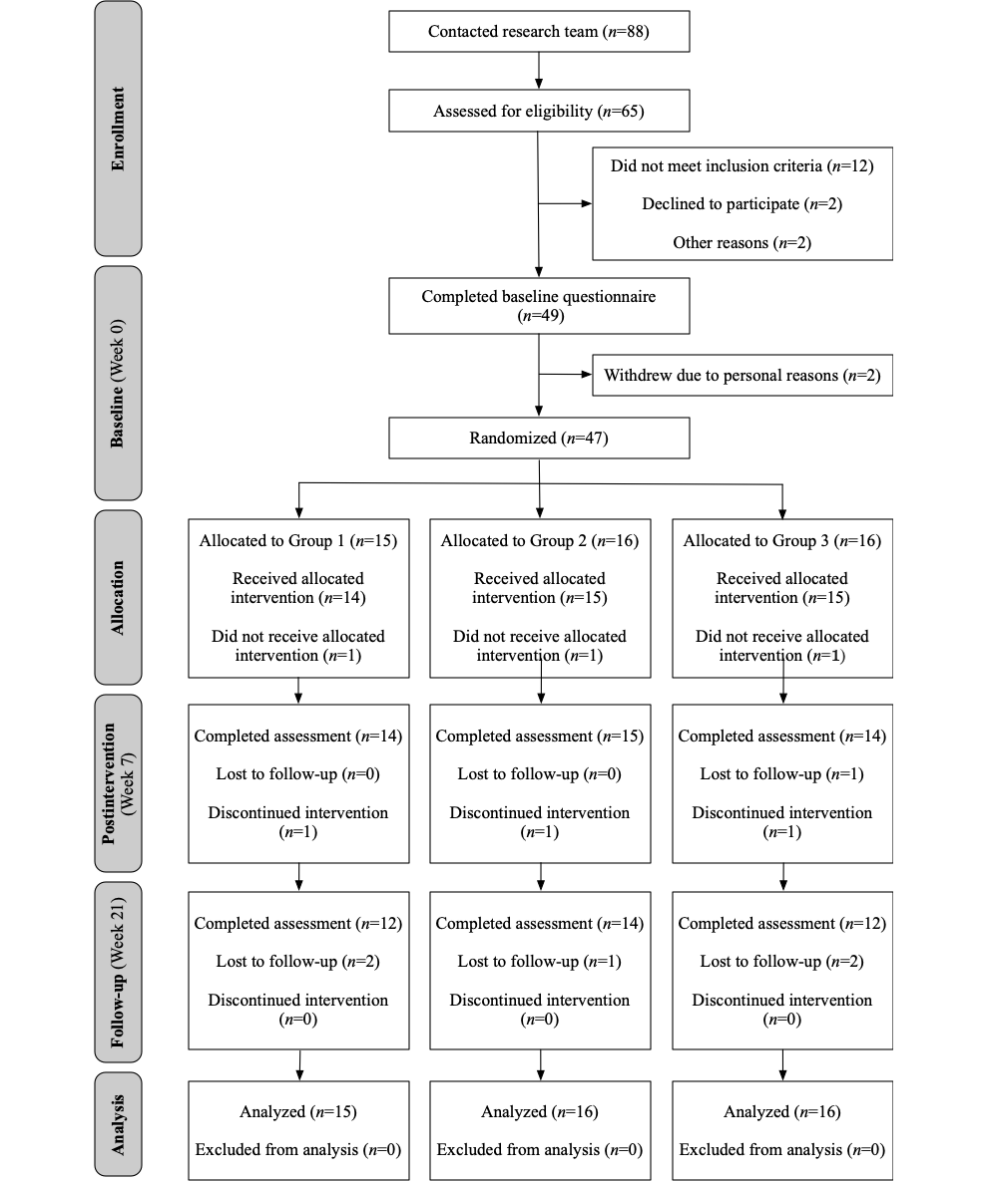
CONSORT (Consolidated Standards of Reporting Trials) flow diagram.

In total, 88 women contacted the first author to express interest in this study. A total of 63 patients were screened for eligibility, of which 49 (78%) provided consent. Of these 49 women, 2 (4%) dropped out after completing the web-based questionnaires but before meeting with the first author in person for anthropometric measurements because of unforeseen time constraints (n=1) and unwillingness to comply with the wearable activity tracker protocol (n=1)*.* The remaining 96% (47/49) of participants were randomized and included in the analyses. Among the participants, 9% (4/47) did not complete the postintervention questionnaires for unspecified reasons (2/4, 50%) or were lost to follow-up (2/4, 40%); an additional 11% (5/47) were lost to follow-up (ie, at the follow-up assessment). A CONSORT flow diagram showing the flow of participants through the trial is shown in Figure 1.

**Figure 2:**
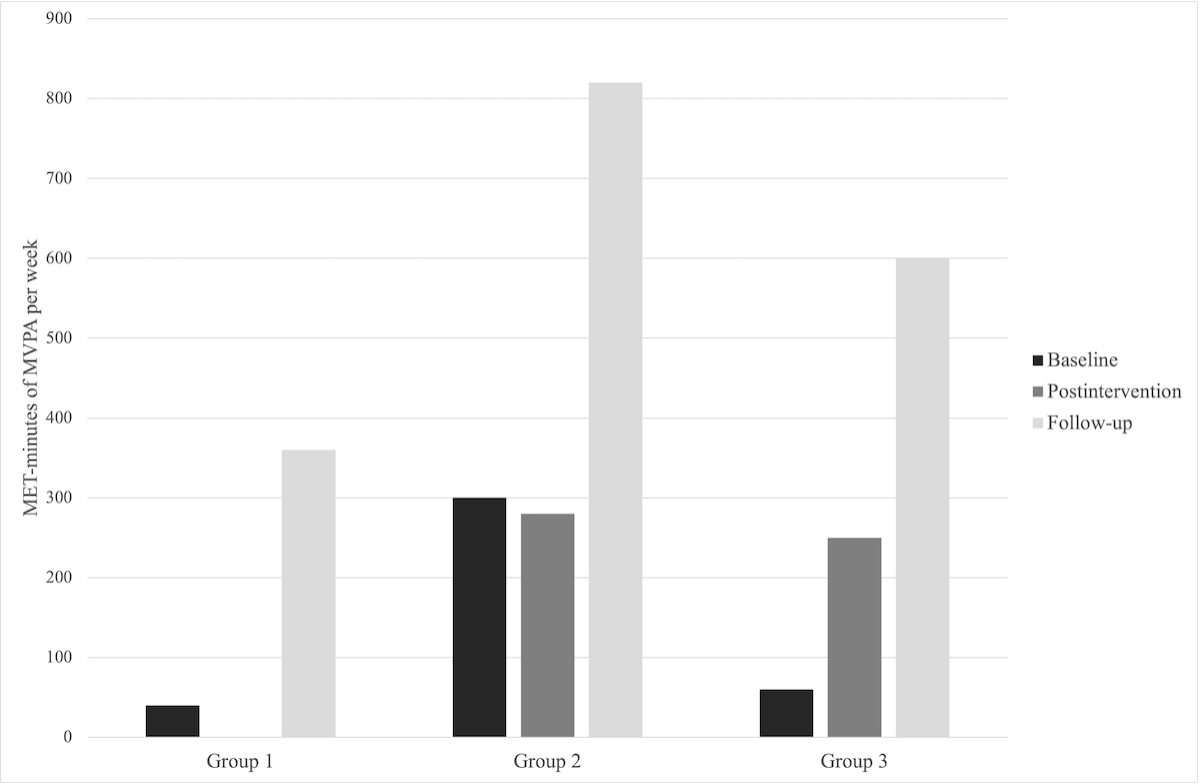
Mean metabolic equivalent of task minutes of moderate to vigorous intensity physical activity per week by group. MET: metabolic equivalent of task; MVPA: moderate to vigorous intensity physical activity.

**Figure 3:**
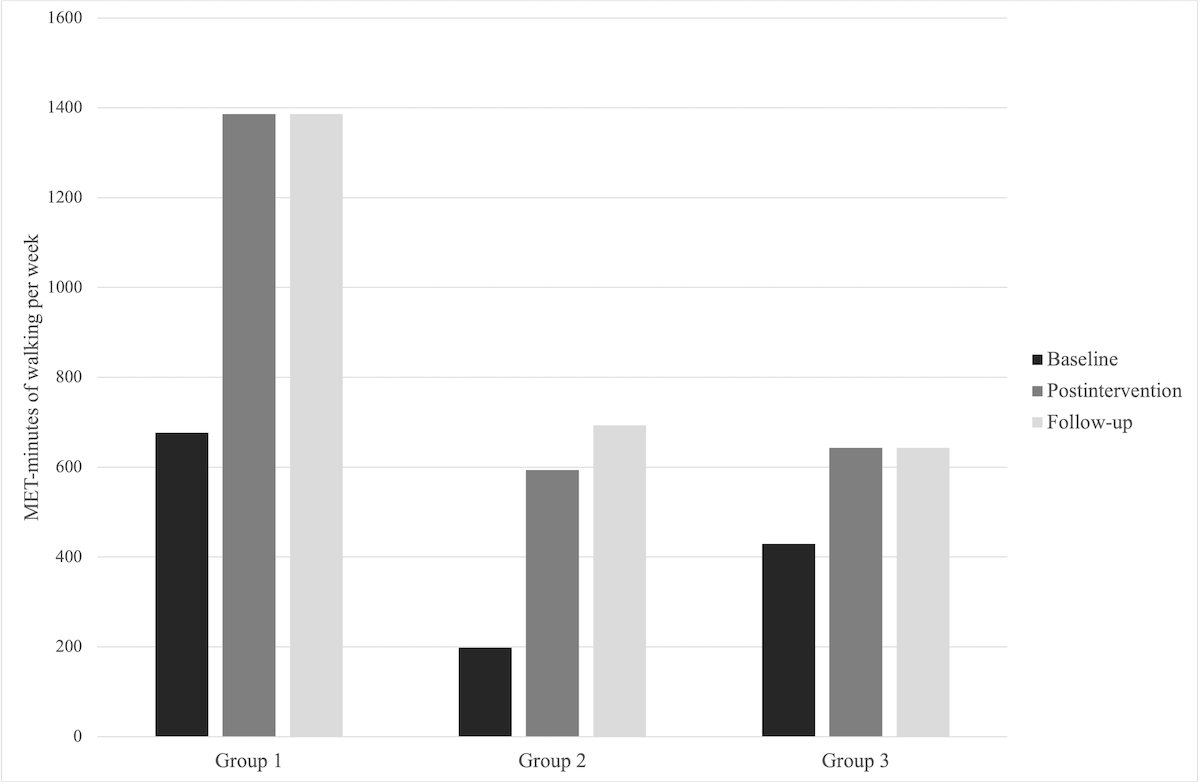
Mean metabolic equivalent of task minutes of walking per week by group. MET: metabolic equivalent of task.

The characteristics of the analytical sample preintervention are presented in Table 1. There were no statistically significant differences found between the groups for the main study variables (ie, PA, basic psychological needs satisfaction, and motivational regulations), age, BMI, or waist circumference. For the 47 participants randomized, the average age was 37.57 years (SD 11.78 years), average BMI was 31.69 kg/m^2^ (SD 5.97 kg/m^2^; with 26/47, 55% classified as obese; BMI ≥30 kg/m^2^), and average waist circumference was 98.54 cm (SD 15.13 cm). Participants reported a history of chronic diseases, including stroke (1/47, 2%), diabetes (3/47, 6%), high blood pressure (7/47, 15%), high cholesterol (5/47, 11%), arthritis (7/47, 15%), asthma (7/47, 15%), and moderate to severe depression (13/47, 28%). The medians and IQRs for all outcome variables are shown in Table 2. Mean MET minutes per week by group for moderate to vigorous intensity PA and walking are presented in Figures 2 and 3, respectively. The effect sizes and 95% CI for the post hoc analyses are presented in Multimedia Appendix 3. Bivariate correlations between change in primary (ie, moderate to vigorous intensity PA and walking) and secondary outcomes (ie, basic psychological needs and motivational regulations) are presented in Multimedia Appendix 4.

**Table 1:** Baseline characteristics of participants randomized (N=47).

Variable		Group 1 (n=15)	Group 2 (n=16)	Group 3 (n=16)	Value, range	
Age (years; n=47), mean (SD)		32.6 (7.8)	39.2 (11.6)	40.6 (14.0)	18-63	
BMI (kg/m^2^; n=47), mean (SD)		31.3 (5.8)	32.9 (5.7)	30.8 (6.5)	23.6-45.7	
Body composition (% fat; n=46), mean (SD)		41.1 (5.8)	42.5 (6.0)	40.1 (7.1)	24.2-55	
Waist circumference (cm; n=47), mean (SD)		97.4 (13.1)	102.8 (16.2)	95.3 (15.7)	71-141	
**Self-rated health (n=47), n (%)**						N/A^a^
	Poor	1 (7)	1 (6)	1 (6)		
	Fair	5 (33)	4 (25)	5 (31)		
	Good	9 (60)	8 (50)	8 (50)		
	Very good	0 (0)	3 (19)	2 (13)		
	Excellent	0 (0)	0 (0)	0 (0)		
**Smoking status (n=47), n (%)**						N/A
	Never smoked	13 (87)	6 (38)	14 (88)		
	Previously smoked	1 (7)	5 (31)	1 (6)		
	Currently smokes	1 (7)	5 (31)	1 (6)		
**Education (n=47), n (%)**						N/A
	High school	0 (0)	1 (6)	1 (6)		
	Some college or university	1 (7)	4 (25)	4 (25)		
	College or university	13 (87)	7 (44)	10 (63)		
	Graduate degree	1 (7)	4 (25)	1 (6)		
**Employment status (n=46), n (%)**						
	Unemployed	2 (13)	3 (19)	4 (25)		
	Student	3 (20)	1 (6)	2 (13)		
	Part-time worker	3 (20)	1 (6)	3 (19)		
	Full-time worker	7 (47)	10 (63)	7 (44)		
**Annual household income (CAD $; US $), n (%)**						N/A
	≤49,999 (34,999)	6 (40)	6 (38)	4 (25)		
	50,000-99,999 (35,000-69,999)	3 (20)	5 (31)	2 (12)		
	>100,000 (70,000)	4 (27)	5 (31)	4 (25)		
**Race (n=47), n (%)**						N/A
	White	11 (73)	14 (88)	14 (88)		
	Other	4 (27)	2 (13)	2 (13)		

^a^N/A indicates the value is not applicable, as the data are presented as number and frequency.

**Table 2:** Median and IQR for all outcome variables.

Variables		Group 1					Group 2					Group 3			
		Preintervention, median (IQR)	Postintervention, median (IQR)	Follow-up, median (IQR)	*P* value	Preintervention, median (IQR)		Postintervention, median (IQR)	Follow-up, median (IQR)	*P* value	Preintervention, median (IQR)		Postintervention, median (IQR)	Follow-up, median (IQR)	*P* value
**Physical activity^a^**															
	MVPA^a,b^	40.00 (720.00)	0.00 (832.00)	360.00 (800.00)	.24	300.00 (480.00)		280.00 (1140.00)	820.00 (2040.00)	.24	60.00 (440.00)		250.00 (920.00)	600.00 (1890.00)	.34
	Walking	676.50 (792.00)	1386.00 (1798.00)	1386.00 (2178.00)	.02^c^	198.00(903.38)		594.00 (2557.50)	693.00 (1311.75)	.162	429.00 (1641.75)		643.00 (2165.63)	643.5.00 (928.13)	.49
**Basic psychological needs satisfaction**															
	Autonomy	5.50 (1.83)	5.17 (1.00)	5.67 (2.00)	.27	4.83 (1.83)		5.33 (1.46)	5.00 (2.08)	.03^c^	4.83 (1.71)		5.83 (1.13)	5.67 (1.83)	.005^c^
	Competence	3.67 (2.83)	4.00 (0.83)	4.00 (1.17)	.02^c^	3.67 (1.71)		3.75 (1.58)	4.00 (3.08)	.34	3.75 (1.25)		3.67 (3.13)	3.42 (2.33)	.55
	Relatedness	3.00 (2.50)	3.83 (2.50)	3.89 (2.67)	.01^c^	3.29 (2.35)		3.75 (2.58)	3.33 (2.21)	.50	3.67 (1.25)		2.83 (3.83)	3.83 (3.00)	.92
**Motivational regulations**															
	Amotivation	0.00 (1.00)	0.00 (0.75)	0.00 (0.75)	.67	0.00 (0.50)		0.00 (0.44)	0.12 (0.94)	.70	0.38 (0.94)		0.38 (1.19)	0.12 (0.90)	.80
	External	1.00 (2.00)	0.50 (2.25)	0.25 (1.50)	.20	0.75 (1.25)		0.50 (1.25)	0.62 (1.19)	.87	1.00 (0.90)		1.00 (1.19)	0.75 (2.00)	.77
	Introjected	2.50 (0.75)	2.00 (1.75)	2.00 (1.00)	.12	2.62 (1.00)		2.50 (0.88)	2.25 (0.94)	.42	2.12 (2.38)		2.00 (2.56)	1.88 (3.19)	.56
	Identified	2.50 (1.00)	2.75 (1.25)	2.50 (1.50)	.59	2.62 (1.00)		2.62 (1.13)	2.25 (1.38)	.09	2.25 (1.38)		2.25 (1.50)	2.12 (2.00)	.86
	Integrated	2.00 (0.75)	2.00 (1.50)	2.00 (1.25)	.63	1.50 (1.31)		2.00 (1.69)	1.75 (1.44)	.77	1.75 (1.88)		1.88 (2.06)	1.88 (1.81)	.92
	Intrinsic	2.25 (1.00)	2.75 (1.50)	2.75 (1.50)	.28	2.25 (1.44)		2.62 (1.44)	2.50 (1.38)	.32	2.12 (1.69)		2.12 (2.25)	2.25 (1.69)	.56

^a^Current physical activity guidelines recommend at least 150 minutes of moderate intensity aerobic PA or at least 75 minutes of vigorous intensity aerobic PA, which is equivalent to at least 450 metabolic equivalent of task (MET) minutes per week to meet PA. PA has been shown to have a dose-response relationship with subsequent health-benefits, therefore higher MET minute scores are considered better.

^b^Moderate to vigorous intensity physical activity.

^c^Indicates a significant within-group difference (*P*<.05).

### PA Behavior

There was a significant increase in MET minutes per week of walking within group 1 (*χ*^2^_2_=7.5; *P*=.02) but not in group 2 (*P*=.16) or group 3 (*P*=.49). Pairwise comparisons using the Wilcoxon signed-rank test revealed that walking increased for group 1 from preintervention to postintervention (*t*_1_=−0.87; *r*=0.16; *P*=.05) although not statistically significant with the Bonferroni correction, and there were no significant changes in walking from preintervention to follow-up (*P*=.25) or from postintervention to follow-up (*P*=.99). In addition, there were no significant changes in moderate to vigorous intensity PA within group 1 (*P*=.24), group 2 (*P*=.24), or group 3 (*P*=.34). There were no significant differences in change scores between the groups for either outcome from preintervention to postintervention (walking, *P*=.26; moderate to vigorous intensity PA, *P*=.40), preintervention to follow-up (walking, *P*=.43; moderate to vigorous intensity PA, *P*=.40), or postintervention to follow-up (walking, *P*=.98; moderate to vigorous intensity PA, *P*=.97).

### PA-Related Basic Psychological Need Satisfaction

A significant change in perceived autonomy was observed within group 2 (*χ*^2^_2_=7.0; *P*=.03) and group 3 (*χ*^2^_2_=10.6; *P*=.005). Pairwise comparisons indicated that perceived autonomy decreased from postintervention to follow-up in group 2 (*t*_1_=0.78; *r*=−0.39; *P*=.08) but not from preintervention to postintervention (*P*=.65) or from preintervention to follow-up (*P*=.99). In addition, perceived autonomy increased significantly from preintervention to postintervention in group 3 (*t*_1_=−1.00; *r*=0.50; *P*=.02) but not from preintervention to follow-up (*P*=.28) or from postintervention to follow-up (*P*=.75).

There was also a significant difference in change between the groups from preintervention to postintervention (H_2_=5.99; *P*=.05) and from postintervention to follow-up (H_2_=6.70; *P*=.04) but not from preintervention to follow-up (*P*=.09). Pairwise comparisons with adjusted *P* values showed that group 3 had a greater increase in perceived autonomy from preintervention to postintervention than group 1 (*P*=.04), but there were no significant differences between groups 1 and 2 (*P*=.67) or groups 2 and 3 (*P*=.63). In addition, from postintervention to follow-up, there was a smaller decrease in perceived autonomy in group 1 compared with group 2 (*P*=.03) but no significant differences between group 1 and group 3 (*P*=.56) or group 2 and group 3 (*P*=.59).

A significant change in perceived competence was observed within group 1 (*χ*^2^_2_=7.6; *P*=.02). Pairwise comparisons revealed a significant increase from preintervention to follow-up (T=−0.90; *r*=.45; *P*=.04) but not from preintervention to postintervention (*P*=.30) or from postintervention to follow-up (*P*=.99). No significant changes in perceived competence were observed in group 2 (*P*=.34) or group 3 (*P*=.55), and there were no significant differences between the groups for change in perceived competence from preintervention to postintervention (*P*=.76), preintervention to follow-up (*P*=.10), or postintervention to follow-up (*P*=.34).

A significant change in perceived relatedness was observed in group 1 (*χ*^2^_2_=8.7; *P*=.01). Pairwise comparisons revealed a significant increase from preintervention to follow-up (T=−0.93; *r*=0.47; *P*=.03) but not from preintervention to postintervention (*P*=.20) or from postintervention to follow-up (*P*=.99). No significant changes in perceived relatedness were found within group 2 (*P*=.50) or group 3 (*P*=.92), and there were no significant differences between the groups for change in perceived relatedness preintervention to postintervention (*P*=.28), preintervention to follow-up (*P*=.07), or postintervention to follow-up (*P*=.15).

### Motivational Regulations for PA

There were no significant changes in amotivation, external, introjected, identified, integrated, or intrinsic motivational regulations for PA within groups across time points (Table 2). There were no significant differences between the groups for changes in any of the motivational regulations for PA. Specifically, there was no significant difference between groups for amotivation (preintervention to postintervention, *P*=.72; preintervention to follow-up, *P*=.75; or postintervention to follow-up, *P*=.67); external (preintervention to postintervention, *P*=.73; preintervention to follow-up, *P*=.42; or postintervention to follow-up, *P*=.67); introjected (preintervention to postintervention, *P*=.60; preintervention to follow-up, *P*=.48; or postintervention to follow-up, *P*=.82); identified (preintervention to postintervention, *P*=.80; preintervention to follow-up, *P*=.21; or postintervention to follow-up, *P*=.30); integrated (preintervention to postintervention, *P*=.95; preintervention to follow-up, *P*=.74; or postintervention to follow-up, *P*=.99); or introjected regulations (preintervention to postintervention, *P*=.60; preintervention to follow-up, *P*=.35; or postintervention to follow-up, *P*=.22).

## Discussion

### Principal Findings

Low PA among women who are overweight and obese is a cause for concern, considering the numerous health benefits associated with regular engagement in PA [6]. The principal finding of this study indicates that adding email behavioral support to a wearable activity tracker intervention yielded an increase in walking of 709.50 MET minutes per week over the course of the 6-week intervention, though such increases were not statistically significantly more than providing women with a wearable activity tracker without emails (*M*_change_=600.19 MET minutes) or only providing them with a copy of PA guidelines (*M*_change_=109.31 MET minutes). Furthermore, no significant changes in moderate to vigorous intensity PA were observed in any of the groups. The findings also show that increases in autonomy preintervention to postintervention were greater for group 2 and 3 participants than for group 1 participants, that perceptions of autonomy decreased toward preintervention values from postintervention to follow-up for group 2, and that perceptions of competence and relatedness increased from preintervention to follow-up for group 1, although increases were not significantly greater than for groups 2 or 3. Collectively, the findings from this study suggest that the autonomy-supportive email intervention received by group 1 could help to promote walking among women who are overweight or obese, but further research is necessary to confirm the current results and perhaps optimize the intervention.

### Results in the Context of Other Literature

Previous studies suggest that, in general, behavior change interventions (including those delivered by email) may be effective in increasing walking among people who are sedentary [50]. The increase in weekly walking observed from preintervention to postintervention for group 1 participants, though not statistically significantly different from the increases observed in groups 2 and 3, supports this assertion. Walking was incorporated into the examples provided in the emails to the participants given evidence suggesting engaging in low-intensity PA (including low-intensity walking) in lieu of sedentary activity may confer meaningful health benefits [29], and may be more accessible and enjoyable for women who are sedentary and overweight or obese. For example, the following excerpt was included in week 2 email:

Walking is an excellent and accessible way to be physically active and improve your health. Running is a higher intensity activity that is also a good choice. The important thing is that you are moving, doing something you enjoy, and that your physical activity choices fit with your lifestyle. Walking is a great way to do this.

In addition, the increase in weekly walking observed in group 1 and group 2 may be explained by the provision of a wearable activity tracker. Consistent with previous studies [12,13], providing participants in both groups with a tracker may have fostered self-monitoring, encouraging participants to walk more each week. Finally, providing participants in all 3 groups with a copy and verbal explanation of the PA guidelines may have contributed to the observed increases. Despite no additional counseling component (for groups 2 and 3), it is possible that receiving the PA guidelines prompted participants to reflect on their current PA behavior and make changes accordingly, and since walking can be easily incorporated into many activities of daily living, it may have been an option for them as they worked toward meeting PA guidelines.

In contrast to research showing that autonomy-supportive interventions and wearable activity tracker interventions can increase moderate to vigorous intensity PA levels [7-10,13], no significant changes in moderate to vigorous intensity PA were observed in this study, regardless of group allocation. Across groups, participants’ ability to increase their moderate to vigorous intensity PA levels, which can be more difficult to incorporate into activities of daily living than walking, may have been hindered by commonly reported barriers (eg, inflexible work schedules, long working hours, household responsibilities and chores, weather conditions, and other commitments) [51,52]. Furthermore, although emails attempted to help group 1 participants overcome such barriers, there remains a need for further improvement. Possibly, 1-way message delivery from the first author to the participants may be insufficient. If so, incorporating 2-way interactive counseling by adding a counseling component that could be delivered over the phone or via videoconferencing to help participants identify and overcome barriers may help to achieve the desired increases in moderate to vigorous intensity PA levels. Another possible explanation for the nonsignificant finding across groups is related to the measure used to assess PA behavior. The IPAQ Short Form is a self-report measure, and people tend to overestimate their PA behavior [53]. In addition, when completing the IPAQ Short Form, participants were asked to provide the amount of time spent per week in vigorous intensity PA, moderate intensity PA, and walking. To ensure consistency of interpretation, explanations of the types of activities included in each category were provided within the questionnaire. However, walking may incur a higher energy expenditure for women who are overweight or obese than for those who are normal weight [54]; therefore, increases in moderate to vigorous intensity walking may have consequently been captured in the walking variables rather than in the moderate to vigorous intensity PA variable. In the future, it may be beneficial to add objective measures of PA (eg, accelerometers) to assess PA intensity (ie, light, moderate, or vigorous) and energy expenditure alongside self-report to capture PA context (ie, work, leisure, transportation, exercise, or walking) to delineate the effects of interventions on different PA outcomes.

A significant increase in perceptions of competence and relatedness for PA was observed in group 1, though not significantly more than in groups 2 and 3, and this may be related to the increases observed in walking. Although PA enjoyment was not assessed in this study, other studies have shown that low-intensity PA such as low-intensity walking can be enjoyable for women who are overweight or obese [30,31], and it can be performed by most women. As such, participants who engaged in walking may have had higher perceptions of competence to engage in PA because they enjoyed it and did not find it hard to do as a result. It is also possible that participants who increased their walking did so in the company of others (eg, friends, spouse, or coworkers) providing opportunities for them to connect and bond with others, and thus increase their perceptions of relatedness. Indeed, some of the content within the emails may have prompted them to do so, as exemplified by the recurring suggestion of *going for a walk with a friend or family member*. Future research should consider how PA context (eg, type of activity, presence of PA companions, or location) is related to basic psychological needs satisfaction, motivational regulations, and PA behavior among women who are overweight or obese.

Moreover, perceived autonomy increased from preintervention to postintervention in groups 2 and 3, and the observed increases were greater than those observed in group 1. Despite no additional behavioral support for groups 2 and 3, simply participating in the study and receiving a copy and verbal explanation of the PA guidelines may have prompted participants to reflect on their PA behavior and consider options for making changes on their own. In addition, consistent with previous studies [12,13], providing group 2 with a wearable activity tracker may have enabled self-monitoring, empowering them to increase their PA levels independently, however they wished to. For group 1, despite recruitment materials providing a description of the intervention, it is possible that participants in group 1 anticipated receiving more support from the facilitator than was provided. Although the emails featured various established motivational and behavior change techniques [23,55], the absence of reciprocal interaction with a facilitator may have limited the amount of autonomy support that could be derived from the emails. Techniques such as encouraging participants to ask questions, using demonstrations, using empathetic listening, providing opportunities for ongoing support, and offering clear, constructive, and relevant feedback were absent from this intervention because of its asynchronous nature. These techniques may provide participants with the support they need to feel confident about their own choices, which in turn could help to increase their perceptions of autonomy. An improvement to the current intervention may be a mix of emails and synchronous sessions through a web-based platform with a specialist who is present in real time and can emphasize certain autonomy-supportive techniques in a personally relevant manner [56,57]. Because of the positive association observed between perceptions of autonomy and PA in previous studies [21], it is critical to determine if such revisions can lead to gains in perceived autonomy.

Finally, unlike Silva et al [9], who found significantly higher introjected, identified, and intrinsic regulations in the intervention group following a 12-month behavior change intervention for PA, no changes were observed in this study for motivational regulations for PA. Levels of amotivation and external regulation were relatively low preintervention, which is consistent with the possibility that the convenience sample of women recruited to this study was, in general, motivated to make changes in their PA behavior. Although participants’ stage of change related to PA was not assessed in this study, it is possible that most were, at minimum, in the contemplation (ie, thinking about change, not yet engaging in change) or preparation stage of change (ie, intending to change in the next 6 months) at the start of the study, given the study eligibility criteria. In this sense, those in the precontemplation stage (ie, unwilling to change or not aware of a problem), which shares features of amotivation, may not have signed up for this study that was focused on promoting PA. Regardless, low levels of, and limited variance in, amotivation and external regulation preintervention likely made it impossible to observe hypothesized decreases in these variables within the current sample. Although the lack of increase in the remaining motivational regulations is unexpected, the maintenance of preintervention moderate levels, particularly among identified regulation and intrinsic motivation, is very important because endorsement of these motivational regulations is associated with higher PA adherence [21]. It is recommended to use qualitative research in future trials to identify reasons associated with the lack of change in motivational regulations and seek out strategies to improve autonomous regulations.

### Limitations

The limitations of this study include the sample size for examining secondary outcomes, possible selection bias toward women who volunteered to participate in this study who may have been more motivated to increase their PA than the general population, and the use of *physical activity* in the stems of the questionnaires used to assess basic psychological needs satisfaction and motivational regulations (rather than *physical activity* and *walking*). The use of self-report to assess PA, although necessary for reasons of feasibility and to avoid the risk of a Hawthorne effect, is also a limitation. Finally, data on whether participants read the emails, and if so, how many times and for how long, were not collected; these use data may have provided valuable information regarding fidelity to guide improvements to the intervention. Relatedly, participants allocated to groups 1 and 2 were asked to self-report how often they wore the wearable activity tracker and how often they looked at their PA data (on their wrist and through the web application); however, the accuracy of these self-report data could not be verified. Thus, although seemingly high as of the 31 participants who received a wearable activity tracker, 79% (n=23) reported wearing their tracker >4 days per week for >12 hours per day, underuse may have led to an underestimation of the effects.

### Conclusions

This study represents an important step toward developing interventions that promote PA among women who are overweight and obese. It sought to evaluate the additional benefit of adding email counseling to a wearable activity tracker intervention (with the provision of PA guidelines). The findings suggest that, among the 3 interventions tested, providing women with behavior support weekly emails in addition to a wearable activity tracker and PA guidelines may help to increase walking behavior over a 6-week period. However, this study has also revealed possible areas for improvement as there were no significant increases in moderate to vigorous intensity PA observed and providing participants with weekly behavior support emails did not foster basic psychological needs satisfaction and motivational regulations for PA over time. These issues need to be addressed in future trials, possibly by adding synchronous sessions with a PA specialist to the weekly behavior support emails and the wearable activity tracker intervention.

## Appendix

Multimedia Appendix 1 Overview of weekly emails.

Multimedia Appendix 2 Properties of all outcome variables.

Multimedia Appendix 3 Effect sizes and 95% CI for all outcome variables for each group.

Multimedia Appendix 4 Pairwise correlations between change scores in moderate-to-vigorous intensity physical activity, walking, basic psychological needs satisfaction, and motivational regulations.

Multimedia Appendix 5 CONSORT-eHEALTH checklist (V 1.6.1).

## Abbreviations

BREQ-3: Behavioral Regulation in Exercise Questionnaire, version 3
IPAQ: International Physical Activity Questionnaire
MET: metabolic equivalent of task
PA: physical activity
PNSE: Psychological Need Satisfaction in Exercise

## References

[1] (2021). Obesity and overweight: key facts. World Health Organization.

[2] Chooi YC, Ding C, Magkos F (2019). The epidemiology of obesity. Metabolism.

[3] King NA, Hopkins M, Caudwell P, Stubbs RJ, Blundell JE (2009). Beneficial effects of exercise: shifting the focus from body weight to other markers of health. Br J Sports Med.

[4] (2020). Physical Activity, Sedentary Behaviour and Sleep (PASS) indicators data tool, 2020 edition. Center for Surveillance and Applied Research, Public Health Agency of Canada.

[5] Colley RC, Garriguet D, Janssen I, Craig CL, Clarke J, Tremblay MS (2011). Physical activity of Canadian adults: accelerometer results from the 2007 to 2009 Canadian Health Measures Survey. Health Rep.

[6] Warburton DE, Bredin SS (2017). Health benefits of physical activity: a systematic review of current systematic reviews. Curr Opin Cardiol.

[7] Edmunds J, Ntoumanis N, Duda JL (2008). Testing a self-determination theory-based teaching style intervention in the exercise domain. Eur J Soc Psychol.

[8] Moustaka F, Vlachopoulos S, Kabitsis C, Theodorakis Y (2012). Effects of an autonomy-supportive exercise instructing style on exercise motivation, psychological well-being, and exercise attendance in middle-age women. J Phys Act Health.

[9] Silva MN, Vieira PN, Coutinho SR, Minderico CS, Matos MG, Sardinha LB, Teixeira PJ (2010). Using self-determination theory to promote physical activity and weight control: a randomized controlled trial in women. J Behav Med.

[10] Silva MN, Markland D, Vieira PN, Coutinho SR, Carraça EV, Palmeira AL, Minderico CS, Matos MG, Sardinha LB, Teixeira PJ (2010). Helping overweight women become more active: need support and motivational regulations for different forms of physical activity. Psychol Sport Exerc.

[11] Cadmus-Bertram LA, Marcus BH, Patterson RE, Parker BA, Morey BL (2015). Randomized trial of a Fitbit-based physical activity intervention for women. Am J Prev Med.

[12] Lewis ZH, Lyons EJ, Jarvis JM, Baillargeon J (2015). Using an electronic activity monitor system as an intervention modality: a systematic review. BMC Public Health.

[13] Brickwood K, Watson G, O'Brien J, Williams AD (2019). Consumer-based wearable activity trackers increase physical activity participation: systematic review and meta-analysis. JMIR Mhealth Uhealth.

[14] Gaudet J, Gallant F, Bélanger M (2017). A Bit of Fit: minimalist intervention in adolescents based on a physical activity tracker. JMIR Mhealth Uhealth.

[15] Hermsen S, Moons J, Kerkhof P, Wiekens C, De GM (2017). Determinants for sustained use of an activity tracker: observational study. JMIR Mhealth Uhealth.

[16] Karapanos E, Gouveia R, Hassenzahl M, Forlizzi J (2016). Wellbeing in the making: peoples’ experiences with wearable activity trackers. Psych Well-Being.

[17] Attig C, Franke T (2019). I track, therefore I walk – Exploring the motivational costs of wearing activity trackers in actual users. Int J Hum-Comput Stud.

[18] Kerner C, Goodyear VA (2017). The motivational impact of wearable healthy lifestyle technologies: a self-determination perspective on fitbits with adolescents. Am J Health Edu.

[19] Mendoza JA, Baker KS, Moreno MA, Whitlock K, Abbey-Lambertz M, Waite A, Colburn T, Chow EJ (2017). A Fitbit and Facebook mHealth intervention for promoting physical activity among adolescent and young adult childhood cancer survivors: A pilot study. Pediatr Blood Cancer.

[20] Ryan RM, Deci EL (2000). Self-determination theory and the facilitation of intrinsic motivation, social development, and well-being. Am Psychol.

[21] Teixeira PJ, Carraça EV, Markland D, Silva MN, Ryan RM (2012). Exercise, physical activity, and self-determination theory: a systematic review. Int J Behav Nutr Phys Act.

[22] Robinson SA, Bisson AN, Hughes ML, Ebert J, Lachman ME (2019). Time for change: using implementation intentions to promote physical activity in a randomised pilot trial. Psychol Health.

[23] Teixeira PJ, Marques MM, Silva MN, Brunet J, Duda JL, Haerens L, La Guardia J, Lindwall M, Lonsdale C, Markland D, Michie S, Moller AC, Ntoumanis N, Patrick H, Reeve J, Ryan RM, Sebire SJ, Standage M, Vansteenkiste M, Weinstein N, Weman-Josefsson K, Williams GC, Hagger MS (2020). A classification of motivation and behavior change techniques used in self-determination theory-based interventions in health contexts. Motiv Sci.

[24] Sullivan AN, Lachman ME (2016). Behavior change with fitness technology in sedentary adults: a review of the evidence for increasing physical activity. Front Public Health.

[25] Ellis DA, Piwek L (2018). Failing to encourage physical activity with wearable technology: what next?. J R Soc Med.

[26] Silva MN, Markland D, Carraça EV, Vieira PN, Coutinho SR, Minderico CS, Matos MG, Sardinha LB, Teixeira PJ (2011). Exercise autonomous motivation predicts 3-yr weight loss in women. Med Sci Sports Exerc.

[27] Ryan R, Deci E (2018). Self-Determination Theory: Basic Psychological Needs in Motivation, Development, and Wellness.

[28] Hall KS, Hyde ET, Bassett DR, Carlson SA, Carnethon MR, Ekelund U, Evenson KR, Galuska DA, Kraus WE, Lee I, Matthews CE, Omura JD, Paluch AE, Thomas WI, Fulton JE (2020). Systematic review of the prospective association of daily step counts with risk of mortality, cardiovascular disease, and dysglycemia. Int J Behav Nutr Phys Act.

[29] Colley RC, Michaud I, Garriguet D (2018). Reallocating time between sleep, sedentary and active behaviours: associations with obesity and health in Canadian adults. Health Rep.

[30] Krinski K, Machado DG, Lirani LS, DaSilva SG, Costa EC, Hardcastle SJ, Elsangedy HM (2017). Let's walk outdoors! Self-paced walking outdoors improves future intention to exercise in women with obesity. J Sport Exerc Psychol.

[31] Decker ES, Ekkekakis P (2017). More efficient, perhaps, but at what price? Pleasure and enjoyment responses to high-intensity interval exercise in low-active women with obesity. Psychol Sport Exerc.

[32] Toner J (2018). Exploring the dark-side of fitness trackers: normalization, objectification and the anaesthetisation of human experience. Perform Enhanc Health.

[33] Schulz KF, Altman DG, Moher D, CONSORT Group (2010). CONSORT 2010 statement: updated guidelines for reporting parallel group randomised trials. Br Med J.

[34] Eysenbach G, CONSORT- E (2011). CONSORT-EHEALTH: improving and standardizing evaluation reports of Web-based and mobile health interventions. J Med Internet Res.

[35] Michie S, Abraham C, Whittington C, McAteer J, Gupta S (2009). Effective techniques in healthy eating and physical activity interventions: a meta-regression. Health Psychol.

[36] Patrick H, Williams GC (2012). Self-determination theory: its application to health behavior and complementarity with motivational interviewing. Int J Behav Nutr Phys Act.

[37] Kayser JW, Cossette S, Alderson M (2014). Autonomy-supportive intervention: an evolutionary concept analysis. J Adv Nurs.

[38] Lewis BA, Napolitano MA, Buman MP, Williams DM, Nigg CR (2017). Future directions in physical activity intervention research: expanding our focus to sedentary behaviors, technology, and dissemination. J Behav Med.

[39] Ridgers ND, McNarry MA, Mackintosh KA (2016). Feasibility and effectiveness of using wearable activity trackers in youth: a systematic review. JMIR Mhealth Uhealth.

[40] Faul F, Erdfelder E, Lang A, Buchner A (2007). G*Power 3: a flexible statistical power analysis program for the social, behavioral, and biomedical sciences. Behav Res Methods.

[41] Bravata DM, Smith-Spangler C, Sundaram V, Gienger AL, Lin N, Lewis R, Stave CD, Olkin I, Sirard JR (2007). Using pedometers to increase physical activity and improve health: a systematic review. J Am Med Assoc.

[42] Wang JB, Cadmus-Bertram LA, Natarajan L, White MM, Madanat H, Nichols JF, Ayala GX, Pierce JP (2015). Wearable sensor/device (Fitbit One) and SMS text-messaging prompts to increase physical activity in overweight and obese adults: a randomized controlled trial. Telemed J E Health.

[43] Ware JE, Sherbourne CD (1992). The MOS 36-ltem Short-Form Health Survey (SF-36): conceptual framework and item selection. Med Care.

[44] (2013). Tanita technial bulletin: body composition measurement repeatability. Tanita.

[45] Craig CL, Marshall AL, Sjöström M, Bauman AE, Booth ML, Ainsworth BE, Pratt M, Ekelund U, Yngve A, Sallis JF, Oja P (2003). International physical activity questionnaire: 12-country reliability and validity. Med Sci Sports Exerc.

[46] Wilson PM, Rogers WT, Rodgers WM, Wild TC (2006). The Psychological Need Satisfaction in Exercise Scale. J Sport Exerc Psychol.

[47] Wilson P, Rodgers W, Loitz C, Scime G (2006). “It's Who I Am … Really!’ The importance of integrated regulation in exercise contexts. J Appl Biobehav Res.

[48] Markland D, Tobin V (2004). A modification to the behavioural regulation in exercise questionnaire to include an assessment of amotivation. J Sport Exerc Psychol.

[49] Wilson PM, Sabiston CM, Mack DE, Blanchard CM (2012). On the nature and function of scoring protocols used in exercise motivation research: an empirical study of the behavioral regulation in exercise questionnaire. Psychol Sport Exerc.

[50] Ogilvie D, Foster CE, Rothnie H, Cavill N, Hamilton V, Fitzsimons CF, Mutrie N, Scottish Physical Activity Research Collaboration (2007). Interventions to promote walking: systematic review. Br Med J.

[51] Eyler AE, Wilcox S, Matson-Koffman D, Evenson KR, Sanderson B, Thompson J, Wilbur J, Rohm-Young D (2002). Correlates of physical activity among women from diverse racial/ethnic groups. J Womens Health Gend Based Med.

[52] Dugan S, Karavolos K, Lynch E, Hollings CS, Fullam F, Lange-Maia BS, Powell LH (2016). A multimethod investigation into physical activity in midlife women. J Phys Act Health.

[53] Sallis JF, Saelens BE (2000). Assessment of physical activity by self-report: status, limitations, and future directions. Res Q Exerc Sport.

[54] Wilms B, Ernst B, Thurnheer M, Weisser B, Schultes B (2014). Correction factors for the calculation of metabolic equivalents (MET) in overweight to extremely obese subjects. Int J Obes (Lond).

[55] Michie S, Ashford S, Sniehotta FF, Dombrowski SU, Bishop A, French DP (2011). A refined taxonomy of behaviour change techniques to help people change their physical activity and healthy eating behaviours: the CALO-RE taxonomy. Psychol Health.

[56] Samdal GB, Eide GE, Barth T, Williams G, Meland E (2017). Effective behaviour change techniques for physical activity and healthy eating in overweight and obese adults; systematic review and meta-regression analyses. Int J Behav Nutr Phys Act.

[57] Gillison FB, Rouse P, Standage M, Sebire SJ, Ryan RM (2019). A meta-analysis of techniques to promote motivation for health behaviour change from a self-determination theory perspective. Health Psychol Rev.

