# Supplementary material for: A Wearable Activity Tracker Intervention With and Without Weekly Behavioral Support Emails to Promote Physical Activity Among Women Who Are Overweight or Obese: Randomized Controlled Trial
Source: JMIR Mhealth Uhealth. 2021 Dec 16;9(12):e28128. doi: 10.2196/28128 (PMC8729328; doi:10.2196/28128)
Supplement: Multimedia Appendix 1 [file mhealth_v9i12e28128_app1.docx]

# Table S1. Overview of weekly emails.

| Week | Purpose | Content | BCTs included  [24] | MBCTs included  [58] | Worksheets |
| --- | --- | --- | --- | --- | --- |
| 1 | Getting motivated for PA: To learn about the benefits and explore motives for PA | Overview of program; definitions, benefits, and recommendations for PA; self-assessment of PA behaviour; personal reasons for making a change; confidence | T1, T16 | MBCTs 1-3, 5-7 | 1.1 Decisional Balance Worksheet  1.2 Importance Ruler  1.3 Confidence Ruler |
| 2 | Exploring PA: To expose the truths behind some physical activity myths and explore personal interests | Benefits of PA; pros and cons of making a change; personal reasons for making a change; myths about PA; choosing interesting and enjoyable types of PA | T1, T18 | MBCTs 3, 5-8, 10 | 2.1 Pros and Cons of physical activity Worksheet  2.2 Exploring physical activity Worksheet |
| 3 | Making a plan and taking action: To build an initial plan to increase PA | Personal reasons for making a change; setting SMART goals; choosing interesting and enjoyable types of PA; writing if/then statements; learning from experience | T5, T7-8, T25 | MBCTs 3, 5-8, 10, 15-17, 19 | 3.1 Action Planning Worksheet |
| 4 | Adjusting your plan: To learn from previous experiences and enhance action plans | Learning from experience; barriers to PA; making small adjustments; self-monitoring; social support | T8, T16, T29 | MBCTs 1, 3, 6-7, 10, 14-17, 19-20 | 4.1 Week-in-review Worksheet  4.2 Barriers to physical activity Information Sheet  4.3 Social Support Worksheet |
| 5 | Maintaining motivation: To learn strategies to help maintain motivation in the face of challenges | Learning from experience; self-monitoring; positive and negative social support; self-talk | T16, T29, T33 | MBCTs 3, 6-8, 10, 14-15, 19-21 | 5.1 Self-monitoring Information Sheet  5.2 Self-talk Worksheet |
| 6 | Keep the momentum going: To review the topics covered throughout the program in preparation to continue making changes independently | Benefits and recommendations for PA; personal reasons for making a change; setting SMART goals; choosing interesting and enjoyable types of PA; writing if/then statements; learning from experience; self-monitoring; social support; long-term thinking | T1, T5, T7-8, T16; T29; T35 | MBCT 1, 3-4, 6-8, 10, 14-17, 19-20 | 6.1 Revised Action Planning Worksheet |

*Notes*. BCTs=Behaviour change techniques. MBCT=Motivation and behaviour change techniques, T=technique.
