# Supplementary material for: A Wearable Activity Tracker Intervention With and Without Weekly Behavioral Support Emails to Promote Physical Activity Among Women Who Are Overweight or Obese: Randomized Controlled Trial
Source: JMIR Mhealth Uhealth. 2021 Dec 16;9(12):e28128. doi: 10.2196/28128 (PMC8729328; doi:10.2196/28128)
Supplement: Multimedia Appendix 2 [file mhealth_v9i12e28128_app2.docx]

**Table S2.** Properties for all outcome variables.

|  |  |  |  |  | Range | |  |
| --- | --- | --- | --- | --- | --- | --- | --- |
| Variable | *n* | Mean | *SD* | α | Potential | Actual | Skew |
| Autonomy |  |  |  |  |  |  |  |
| Baseline | 47 | 4.84 | 1.05 | .880 | 1-6 | 2.67-6.0 | -0.64* |
| Post-intervention | 47 | 5.26 | 0.80 | .763 | 1-6 | 2.83-6.0 | -1.12* |
| Follow-up | 47 | 4.99 | 1.13 | .928 | 1-6 | 2.17-6.0 | -0.95* |
| Competence |  |  |  |  |  |  |  |
| Baseline | 47 | 3.49 | 1.34 | .925 | 1-6 | 1.0-6.0 | -0.05 |
| Post-intervention | 47 | 3.79 | 1.28 | .939 | 1-6 | 1.0-6.0 | -0.59* |
| Follow-up | 47 | 3.69 | 1.37 | .965 | 1-6 | 1.0-6.0 | -0.35* |
| Relatedness |  |  |  |  |  |  |  |
| Baseline | 47 | 3.39 | 1.31 | .903 | 1-6 | 1.0-6.0 | -0.12 |
| Post-intervention | 47 | 3.50 | 1.59 | .948 | 1-6 | 1.0-6.0 | -0.05* |
| Follow-up | 47 | 3.55 | 1.58 | .949 | 1-6 | 1.0-6.0 | 0.04* |
| Amotivation |  |  |  |  |  |  |  |
| Baseline | 47 | 0.47 | 0.73 | .731 | 0-4 | 0-3.00 | 1.84* |
| Post-intervention | 47 | 0.49 | 0.74 | .803 | 0-4 | 0-3.00 | 1.64* |
| Follow-up | 47 | 0.58 | 0.79 | .879 | 0-4 | 0-2.50 | 1.24* |
| External |  |  |  |  |  |  |  |
| Baseline | 47 | 0.99 | 0.80 | .746 | 0-4 | 0-3.0 | 0.56* |
| Post-intervention | 47 | 1.01 | 1.02 | .863 | 0-4 | 0-3.50 | 0.96* |
| Follow-up | 47 | 0.91 | 0.94 | .790 | 0-4 | 0-3.50 | 0.87* |
| Introjected |  |  |  |  |  |  |  |
| Baseline | 47 | 2.34 | 0.97 | .740 | 0-4 | 0-4.0 | -0.66* |
| Post-intervention | 47 | 2.21 | 1.20 | .874 | 0-4 | 0-4.0 | -0.35* |
| Follow-up | 47 | 2.01 | 1.17 | .925 | 0-4 | 0-4.0 | -0.22* |
| Identified |  |  |  |  |  |  |  |
| Baseline | 47 | 2.29 | 0.71 | .648 | 0-4 | 0.25-3.50 | -0.70* |
| Post-intervention | 47 | 2.48 | 0.88 | .789 | 0-4 | 0.50-4.0 | -0.44 |
| Follow-up | 47 | 2.30 | 0.93 | .798 | 0-4 | 0-4.0 | -0.52 |
| Integrated |  |  |  |  |  |  |  |
| Baseline | 47 | 1.67 | 0.81 | .795 | 0-4 | 0-3.0 | -0.28 |
| Post-intervention | 47 | 1.86 | 1.01 | .843 | 0-4 | 0-3.75 | -0.03 |
| Follow-up | 47 | 1.77 | 0.95 | .798 | 0-4 | 0-4.0 | 0.03 |
| Intrinsic |  |  |  |  |  |  |  |
| Baseline | 47 | 2.20 | 0.97 | .893 | 0-4 | 0-3.75 | -0.45 |
| Post-intervention | 47 | 2.31 | 1.08 | .922 | 0-4 | 0-4.0 | -0.62* |
| Follow-up | 47 | 2.22 | 1.08 | .930 | 0-4 | 0-4.0 | -0.72* |
| MVPA (MET-minutes per week) |  |  |  |  |  |  |  |
| Baseline | 47 | 447.44 | 761.26 |  | 0-∞ | 0-2400.0 | 1.82* |
| Post-intervention | 47 | 797.28 | 1486.10 |  | 0-∞ | 0-8640.0 | 3.78* |
| Follow-up | 47 | 1214.04 | 2408.50 |  | 0-∞ | 0-15120.0 | 4.52* |
| Walking (MET-minutes per week) |  |  |  |  |  |  |  |
| Baseline | 47 | 861.16 | 1104.84 |  | 0-∞ | 0-4158.0 | 1.62* |
| Post-intervention | 47 | 1385.30 | 1342.22 |  | 0-∞ | 0-4158.0 | 0.84* |
| Follow-up | 47 | 1239.40 | 1366.75 |  | 0-∞ | 0-4158.0 | 1.22* |

*Notes.* SD=standard deviation. MET = metabolic equivalent of task. * Indicates significant at *P<*.05 based on Shapiro-Wilk test.
