# Supplementary material for: A Wearable Activity Tracker Intervention With and Without Weekly Behavioral Support Emails to Promote Physical Activity Among Women Who Are Overweight or Obese: Randomized Controlled Trial
Source: JMIR Mhealth Uhealth. 2021 Dec 16;9(12):e28128. doi: 10.2196/28128 (PMC8729328; doi:10.2196/28128)
Supplement: Multimedia Appendix 3 [file mhealth_v9i12e28128_app3.docx]

**Table S3.** Effect sizes and 95% CI for all outcome variables for each group.

|  | | | | | | Group 1 | | | | Group 2 | | | | Group 3 | | | |
| --- | --- | --- | --- | --- | --- | --- | --- | --- | --- | --- | --- | --- | --- | --- | --- | --- | --- |
|  | | | | | | Time | *P* value | R | 95% CI | T | *P* value | r | 95% CI | T | *P* value | r | 95% CI |
| **MVPA^a^** | | | | | | | | | | | | | | | | | |
|  | T1^b^ to T2^c^ | | | | | −0.33 | .99 | −0.17 | −1.05 to 0.38 | −0.47 | .56 | −0.24 | −1.16 to 0.22 | −0.34 | .99 | −0.17 | −1.04 to 0.35 |
|  | T2^c^ to T3^d^ | | | | | −0.13 | .99 | −0.07 | −0.85 to 0.58 | 0.00 | .99 | 0.00 | −0.69 to 0.69 | −0.06 | .99 | −0.03 | −0.76 to 0.63 |
|  | T1^b^ to T3^d^ | | | | | −0.47 | .60 | −0.23 | −1.18 to 0.25 | −0.47 | .56 | −0.24 | −1.16 to 0.22 | −0.41 | .75 | −0.20 | −1.1 to 0.29 |
| **Walking** | | | | | | | | | | | | | | | | | |
|  | T1^b^ to T2^c^ | | | | | −0.87^e^ | .05^e^ | −0.43^e^ | −1.58 to −0.15e | −0.63 | .23 | −0.31 | −1.32 to 0.07 | −0.31 | .99 | −0.16 | −1.01 to 0.38 |
|  | T2^c^ to T3^d^ | | | | | 0.23 | .99 | 0.12 | −0.48 to 0.95 | 0.31 | .99 | 0.16 | −0.38 to 1.01 | 0.34 | .99 | 0.17 | −0.35 to 1.04 |
|  | T1^b^ to T3^d^ | | | | | −0.63 | .25 | −0.32 | −1.35 to 0.08 | −0.31 | .99 | −0.16 | −1.01 to 0.38 | 0.03 | .99 | 0.02 | −0.66 to 0.73 |
| **Needs satisfaction** | | | | | | | | | | | | | | | | | |
|  | **Autonomy** | | | | | | | | | | | | | | | | |
|  |  | | T1^b^ to T2^c^ | | | 0.07 | .99 | 0.03 | −0.65 to 0.78 | −0.44 | .65 | −0.22 | −1.13 to 0.25 | −1.00^e^ | .01^e^ | −0.50^e^ | −1.7 to −0.31e |
|  |  | | T2^c^ to T3^d^ | | | −0.43 | .71 | −0.22 | −1.15 to 0.28 | 0.78 | .08 | 0.39 | 0.09 to 1.48 | 0.41 | .75 | 0.20 | −0.29 to 1.1 |
|  |  | | T1^b^ to T3^d^ | | | −0.37 | .95 | −0.18 | −1.08 to 0.35 | 0.34 | .99 | 0.17 | −0.35 to 1.04 | −0.59 | .28 | −0.30 | −1.29 to 0.1 |
|  | **Competence** | | | | | | | | | | | | | | | | |
|  |  | | | | T1^b^ to T2^c^ | −0.60 | .30 | −0.30 | −1.31 to 0.12 | −0.44 | .65 | −0.22 | −1.13 to 0.25 | −0.34 | .99 | −0.17 | −1.04 to 0.35 |
|  |  | | | | T2^c^ to T-3^d^ | −0.30 | .99 | −0.15 | −1.01 to 0.42 | 0.31 | .99 | 0.16 | −0.38 to 1.01 | 0.22 | .99 | 0.11 | −0.47 to 0.91 |
|  |  | | | | T1^b^ to T3^d^ | −0.90^e^ | .04^e^ | −0.45^e^ | −1.61 to −0.18^e^ | −0.12 | .99 | −0.06 | −0.82 to 0.57 | −0.12 | .99 | −0.06 | −0.82 to 0.57 |
|  | **Relatedness** | | | | | | | | | | | | | | | | |
|  |  | | | | T1^b^ to T2^c^ | −0.67 | .20 | −0.33 | −1.38 to 0.05 | −0.28 | .99 | −0.14 | −0.98 to 0.41 | −0.06 | .99 | −0.03 | −0.76 to 0.63 |
|  |  | | | | T2^c^ to T3^d^ | −0.27 | .99 | −0.13 | −0.98 to 0.45 | 0.38 | .87 | 0.19 | −0.32 to 1.07 | −0.12 | .99 | −0.06 | −0.82 to 0.57 |
|  |  | | | | T1^b^ to T3^d^ | −0.93^e^ | .03^e^ | −0.47^e^ | −1.65 to 0.22^e^ | 0.09 | .99 | 0.05 | −0.6 to 0.79 | −0.06 | .99 | −0.03 | −0.76 to 0.63 |
| **Motivational regulations** | | | | | | | | | | | | | | | | | |
|  | **Amotivation** | | | | | | | | | | | | | | | | |
|  |  | | | T1^b^ to T2^c^ | | 0.20 | .99 | 0.10 | −0.51 to 0.92 | 0.06 | .99 | 0.03 | −0.63 to 0.76 | −0.16 | .99 | −0.08 | −0.85 to 0.54 |
|  |  | | | T2^c^ to T3^d^ | | 0.00 | .99 | 0.00 | −0.72 to 0.72 | −0.22 | .99 | −0.11 | −0.91 to 0.47 | 0.12 | .99 | 0.06 | −0.57 to 0.82 |
|  |  | | | T1^b^ to T3^d^ | | 0.20 | .99 | 0.10 | −0.51 to 0.92 | −0.16 | .99 | −0.08 | −0.85 to 0.54 | −0.03 | .99 | −0.02 | −0.73 to 0.66 |
|  | **External** | | | | | | | | | | | | | | | | |
|  |  | | | T1^b^ to T2^c^ | | 0.03 | .99 | 0.02 | −0.68 to 0.75 | 0.16 | .99 | 0.08 | −0.54 to 0.85 | 0.22 | .99 | 0.11 | −0.47 to 0.91 |
|  |  | | | T2^c^ to T3^d^ | | 0.43 | .71 | 0.22 | −0.28 to 1.15 | −0.03 | .99 | −0.02 | −0.73 to 0.66 | −0.06 | .99 | −0.03 | −0.76 to 0.63 |
|  |  | | | T1^b^ to T3^d^ | | 0.47 | .60 | 0.23 | −0.25 to 1.18 | 0.12 | .99 | 0.06 | −0.57 to 0.82 | 0.16 | .99 | 0.08 | −0.54 to 0.85 |
|  | **Introjected** | | | | | | | | | | | | | | | | |
|  | |  | | | T1^b^ to T2^c^ | 0.50 | .51 | 0.25 | −0.22 to 1.22 | −0.22 | .99 | −0.11 | −0.91 to 0.47 | 0.28 | .99 | 0.14 | −0.41 to 0.98 |
|  | |  | | | T2^c^ to T3^d^ | 0.10 | .99 | 0.05 | −0.62 to 0.81 | 0.44 | .65 | 0.22 | −0.25 to 1.13 | 0.00 | .99 | 0.00 | −0.69 to 0.69 |
|  | |  | | | T1^b^ to T3^d^ | 0.60 | .30 | 0.30 | −0.12 to 1.31 | 0.22 | .99 | 0.11 | −0.47 to 0.91 | 0.28 | .99 | 0.14 | −0.41 to 0.98 |
|  | | **Identified** | | | | | | | | | | | | | | | |
|  | |  | | | T1^b^ to T2^c^ | −0.17 | .99 | −0.08 | −0.88 to 0.55 | −0.44 | .65 | −0.22 | −1.13 to 0.25 | −0.16 | .99 | −0.08 | −0.85 to 0.54 |
|  | |  | | | T2^c^ to T3^d^ | −0.17 | .99 | −0.08 | −0.88 to 0.55 | 0.69 | .16 | 0.34 | −0.01 to 1.38 | 0.12 | .99 | 0.06 | −0.57 to 0.82 |
|  | |  | | | T1^b^ to T3^d^ | −0.33 | .99 | −0.17 | −1.05 to 0.38 | 0.25 | .99 | 0.13 | −0.44 to 0.95 | −0.03 | .99 | −0.02 | −0.73 to 0.66 |
|  | | **Integrated** | | | | | | | | | | | | | | | |
|  | |  | | | T1^b^ to T2^c^ | −0.20 | .99 | −0.10 | −0.92 to 0.51 | −0.16 | .99 | −0.08 | −0.85 to 0.54 | −0.12 | .99 | −0.06 | −0.82 to 0.57 |
|  | |  | | | T2^c^ to T3^d^ | −0.10 | .99 | −0.05 | −0.81 to 0.62 | −0.06 | .99 | −0.03 | −0.76 to 0.63 | 0.06 | .99 | 0.03 | −0.63 to 0.76 |
|  | |  | | | T1^b^ to T3^d^ | −0.30 | .99 | −0.15 | −1.01 to 0.42 | −0.22 | .99 | −0.11 | −0.91 to 0.47 | −0.06 | .99 | −0.03 | −0.76 to 0.63 |
|  | | **Intrinsic** | | | | | | | | | | | | | | | |
|  | |  | | | T1^b^ to T2^c^ | −0.40 | .82 | −0.20 | −1.12 to 0.31 | 0.00 | .99 | 0.00 | −0.69 to 0.69 | −0.25 | .99 | −0.13 | −0.95 to 0.44 |
|  | |  | | | T2^c^ to T3^d^ | −0.50 | .51 | −0.25 | −1.22 to 0.22 | 0.38 | .87 | 0.19 | −0.32 to 1.07 | 0.31 | .99 | 0.16 | −0.38 to 1.01 |
|  | |  | | | T1^b^ to T3^d^ | −0.10 | .99 | −0.05 | −0.81 to 0.62 | 0.38 | .87 | 0.19 | −0.32 to 1.07 | 0.06 | .99 | 0.03 | −0.63 to 0.76 |

^a^MVPA: moderate-to-vigorous intensity physical activity.

^b^T1: preintervention or prerandomization.
^c^T2: postintervention.
^d^T3: follow-up.

^e^Indicates significant differences between the groups (*P*<.05).
