# Supplementary material for: A Wearable Activity Tracker Intervention With and Without Weekly Behavioral Support Emails to Promote Physical Activity Among Women Who Are Overweight or Obese: Randomized Controlled Trial
Source: JMIR Mhealth Uhealth. 2021 Dec 16;9(12):e28128. doi: 10.2196/28128 (PMC8729328; doi:10.2196/28128)
Supplement: Multimedia Appendix 4 [file mhealth_v9i12e28128_app4.docx]

**Multimedia Appendix 4**

**Table S1.** Pairwise correlations between change scores from baseline to post-intervention in MVPA, walking, basic psychological needs satisfaction and motivational regulations for total sample.

|  | 1 | 2 | 3 | 4 | 5 | 6 | 7 | 8 | 9 | 10 | 11 |
| --- | --- | --- | --- | --- | --- | --- | --- | --- | --- | --- | --- |
| 1. Δ MVPA | - | -.12 | .01 | .05 | -.11 | -.01 | -.04 | -.01 | **.35** | .10 | .20 |
| 2. Δ Walking |  | - | .01 | -.02 | -.08 | -.20 | ***-.40*** | -.05 | -.27 | -.19 | .05 |
| 3. Δ Autonomy |  |  | - | .24 | .03 | .05 | -.04 | .17 | .02 | .23 | -.07 |
| 4. Δ Competence |  |  |  | - | **.29** | .09 | -.08 | .13 | .26 | .11 | .08 |
| 5. Δ Relatedness |  |  |  |  | - | .19 | .05 | .12 | .14 | .23 | .11 |
| 6. Δ Amotivation |  |  |  |  |  | - | .12 | -.16 | -.01 | **-.31** | **-.29** |
| 7. Δ External |  |  |  |  |  |  | - | .10 | .13 | .15 | -.04 |
| 8. Δ Introjected |  |  |  |  |  |  |  | - | .24 | ***.41*** | .25 |
| 9. Δ Identified |  |  |  |  |  |  |  |  | - | *.53* | ***.54*** |
| 10. Δ Integrated |  |  |  |  |  |  |  |  |  | - | ***.45*** |
| 11. Δ Intrinsic |  |  |  |  |  |  |  |  |  |  | - |

*Notes.* Boldface indicates significance at *P*<.05; Boldface and italics indicates significance at *P*<.01.

**Table S2.** Pairwise correlations between change scores from baseline to post-intervention in MVPA, walking, basic psychological needs satisfaction and motivational regulations for Group 1.

|  | 1 | 2 | 3 | 4 | 5 | 6 | 7 | 8 | 9 | 10 | 11 |
| --- | --- | --- | --- | --- | --- | --- | --- | --- | --- | --- | --- |
| 1. Δ MVPA | - | -.43 | .09 | -.08 | -.33 | -.12 | .31 | -.14 | .34 | **.59** | .50 |
| 2. Δ Walking |  | - | .16 | .14 | .32 | -.16 | ***-.71*** | .20 | -.39 | -.19 | -.01 |
| 3. Δ Autonomy |  |  | - | .51 | .30 | .14 | -.10 | .45 | .17 | .48 | .04 |
| 4. Δ Competence |  |  |  | - | .23 | .19 | .04 | .25 | .34 | .28 | .02 |
| 5. Δ Relatedness |  |  |  |  | - | **.53** | .05 | -.01 | .03 | -.05 | -.24 |
| 6. Δ Amotivation |  |  |  |  |  | - | .48 | -.19 | .08 | -.36 | -.34 |
| 7. Δ External |  |  |  |  |  |  | - | -.05 | .34 | .12 | -.25 |
| 8. Δ Introjected |  |  |  |  |  |  |  | - | .35 | .45 | .41 |
| 9. Δ Identified |  |  |  |  |  |  |  |  | - | .46 | **.56** |
| 10. Δ Integrated |  |  |  |  |  |  |  |  |  | - | .50 |
| 11. Δ Intrinsic |  |  |  |  |  |  |  |  |  |  | - |

*Notes.* Boldface indicates significance at *P*<.05; Boldface and italics indicates significance at *P*<.01.

**Table S3.** Pairwise correlations between change scores from baseline to post-intervention in MVPA, walking, basic psychological needs satisfaction and motivational regulations for Group 2.

|  | 1 | 2 | 3 | 4 | 5 | 6 | 7 | 8 | 9 | 10 | 11 |
| --- | --- | --- | --- | --- | --- | --- | --- | --- | --- | --- | --- |
| 1. Δ MVPA | - | -.07 | .04 | -.07 | -.07 | -.06 | -.04 | .01 | **.56** | .11 | **.62** |
| 2. Δ Walking |  | - | -.12 | -.38 | -.30 | .04 | -.26 | -.40 | -.40 | -.31 | .07 |
| 3. Δ Autonomy |  |  | - | .34 | -.12 | -.08 | -.10 | -.24 | -.05 | .09 | -.19 |
| 4. Δ Competence |  |  |  | - | .18 | .37 | .34 | .26 | .14 | .13 | .06 |
| 5. Δ Relatedness |  |  |  |  | - | -.18 | .37 | .36 | .01 | .28 | .16 |
| 6. Δ Amotivation |  |  |  |  |  | - | -.19 | -.10 | -.09 | -.26 | -.26 |
| 7. Δ External |  |  |  |  |  |  | - | .33 | -.27 | -.04 | .25 |
| 8. Δ Introjected |  |  |  |  |  |  |  | - | .39 | **.53** | .41 |
| 9. Δ Identified |  |  |  |  |  |  |  |  | - | **.61** | **.54** |
| 10. Δ Integrated |  |  |  |  |  |  |  |  |  | - | .40 |
| 11. Δ Intrinsic |  |  |  |  |  |  |  |  |  |  | - |

*Notes.* Boldface indicates significance at *P*<.05; Boldface and italics indicates significance at *P*<.01.

**Table S4.** Pairwise correlations between change scores from baseline to post-intervention in MVPA, walking, basic psychological needs satisfaction and motivational regulations for Group 3.

|  | 1 | 2 | 3 | 4 | 5 | 6 | 7 | 8 | 9 | 10 | 11 |
| --- | --- | --- | --- | --- | --- | --- | --- | --- | --- | --- | --- |
| 1. Δ MVPA | - | -.09 | -.14 | -.15 | -.39 | .46 | -.27 | -.17 | -.35 | **.59** | **.58** |
| 2. Δ Walking |  | - | .18 | -.08 | -.48 | **-.50** | -.13 | .17 | -.01 | -.02 | .09 |
| 3. Δ Autonomy |  |  | - | .06 | .12 | .01 | -.01 | .24 | .01 | .12 | -.10 |
| 4. Δ Competence |  |  |  | - | .32 | -.08 | -.28 | -.26 | .29 | -.07 | .13 |
| 5. Δ Relatedness |  |  |  |  | - | .23 | -.02 | .03 | .31 | .45 | .29 |
| 6. Δ Amotivation |  |  |  |  |  | - | -.16 | -.20 | -.01 | -.34 | -.26 |
| 7. Δ External |  |  |  |  |  |  | - | .12 | .30 | .44 | .04 |
| 8. Δ Introjected |  |  |  |  |  |  |  | - | -.12 | .09 | -.01 |
| 9. Δ Identified |  |  |  |  |  |  |  |  | - | .48 | **.61** |
| 10. Δ Integrated |  |  |  |  |  |  |  |  |  | - | **.53** |
| 11. Δ Intrinsic |  |  |  |  |  |  |  |  |  |  | - |

*Notes.* Boldface indicates significance at *P*<.05; Boldface and italics indicates significance at *P*<.01.
